# In-plane hybrid design and mechanical properties of Ti-6Al-4 V TPMS structures

**DOI:** 10.1038/s41598-026-49318-0

**Published:** 2026-04-17

**Authors:** Yushuang Fang, Shilin Yan, Xiangyu Xie, Cheng Wang, Liang Ke, Xiaonan Zhang, Yongjing Li

**Affiliations:** 1 Hubei Key Laboratory of Theory and Application of Advanced Materials Mechanics, School of Physics and Mechanics, Wuhan University of Technology, Wuhan, 430070 China; 2 School of Mechanical and Electrical Engineering, Wuhan University of Technology, Wuhan, 430070 China; 3 State Key Laboratory of Advanced Technology for Materials Synthesis and Processing, Wuhan University of Technology, Wuhan, 430070 China

**Keywords:** Triply periodic minimal surface, Mechanical properties, Additive manufacturing, Hybrid lattices, Engineering, Materials science

## Abstract

Triply Periodic Minimal Surface (TPMS) porous structures are widely utilized in aerospace, automotive, and medical applications due to their high specific strength, large surface area, and uniform stress distribution. However, current research typically focuses on linear hybridization under unidirectional loading to improve mechanical properties. This approach frequently overlooks performance uncertainties in other loading directions. To address these limitations, this paper introduces an in-plane hybrid design strategy. This strategy aims to enhance mechanical properties and energy absorption capacity under different loading directions. Two novel hybrid configurations combining Gyroid and IWP topologies were fabricated from Ti-6Al-4 V using Laser Powder Bed Fusion (PBF-LB/M), alongside their uniform counterparts. Corresponding finite element models were established for all uniform and hybrid structures to perform quasi-static compression simulations. The specimens were analyzed for manufacturing quality, pore formation, and crack evolution using scanning electron microscopy. Furthermore, quasi-static mechanical properties were evaluated under two distinct loading directions (I and II). The simulated stress-strain curves, deformation patterns, and failure modes were compared with the experimental results to validate the models and clarify the deformation mechanism. Results demonstrate that the in-plane hybrid structures exhibit plateau stresses ranging from 90.87 to 99.90 MPa and specific energy absorption (SEA) values between 44.43 and 58.78 J/g. Compared to the uniform structures, both hybrid designs achieved higher SEA when loaded in direction I, with improvements of 15% and 27%, respectively. Notably, the G-IWP-2-I configuration demonstrated the most superior overall mechanical performance. These findings are expected to advance the application of TPMS structures in lightweight design for the aerospace and automotive engineering fields.

## Introduction

Lattice structures, formed by the periodic arrangement of specific micro-architectural unit cells, possess favorable mechanical, thermal, acoustic, and electromagnetic properties. This has led to their widespread application in fields such as aerospace, defense, and biomedical engineering^1–6^. Lattice structures provide more precise stress transfer, tunable mechanical properties, and greater design freedom, offering significant advantages for lightweight applications^7–13^. Moreover, the rapid development of additive manufacturing has further enabled the fabrication of increasingly complex lattice designs. Their underlying biomimetic principles and energy absorption mechanisms have attracted intense interest from both researchers and industry, leading to seminal papers published in journals such as *Nature* and *Science*^14–17^. As a fundamental category, strut-based lattice structures have been extensively studied and continuously optimized. For instance, through topological optimization such as adding specific reinforcing struts, these structures can achieve effective stress redistribution and significantly enhanced compressive strength and energy absorption capabilities^18,19^.

Despite their potential, conventional strut-based lattice structures present considerable obstacles to industrial adoption. Their fabrication typically necessitates sacrificial supports and, more critically, they are prone to localized stress concentrations under loading, creating a high risk of sudden, catastrophic failure^20–22^. As a promising alternative designed to overcome these issues, Triply Periodic Minimal Surface (TPMS) structures have emerged. TPMS architectures are distinguished by their excellent specific strength and stiffness, coupled with a high specific surface area. These desirable properties are a direct result of their unique topology, which is mathematically defined as a smooth, continuous surface with zero mean curvature, meaning both solid and void phases are fully interconnected without isolated cavities^21–28^.

These inherent advantages have motivated extensive research to quantify the mechanical behavior and failure mechanisms of various TPMS topologies, particularly the Primitive, Gyroid, and Schoen I-Wrapped Package (IWP) structures. The geometric parameters of these structures significantly influence their mechanical properties and energy absorption characteristics. These key parameters include cell size, wall thickness, and topological configuration. For instance, Yan et al.^29–31^ examined the quasi-static compressive properties of Ti-6Al-4 V and Al-Si10-Mg TPMS structures fabricated by Selective Laser Melting (SLM). They established power-law relationships between relative density, Young’s modulus, and specific energy absorption based on the Gibson-Ashby model. Similarly, Zhang et al.^32^ combined experiments and simulations to study the quasi-static mechanical properties of the Primitive structure. By modifying the Gibson-Ashby equation, they developed a unified mathematical model that connects structural parameters, such as thickness and C-value, to mechanical performance. Furthermore, Kadkhodapour et al.^33^ employed finite element analysis to investigate the deformation and failure mechanisms of porous titanium biomaterials with Cubic and Diamond lattice structures. Their results indicated that the tensile-dominated Cubic structure fails in a layer-by-layer pattern, while the bending-dominated Diamond structure forms a distinct 45° shear band. To further improve the compressive performance and energy absorption of TPMS structures, functionally graded designs have been proposed. Structural optimization can generate graded TPMS structures with linearly varying porosity along a specific direction. These designs meet varying local performance requirements. They also effectively enhance mechanical stability and failure resistance. Li et al.^34^ and Maskery et al.^35^ designed graded lattice structures with a continuous relative density gradient along the loading direction. They found that these graded structures absorb more energy per unit weight than uniform lattice structures. Although gradient designs significantly improve mechanical properties in a specific direction, the resulting anisotropy may weaken performance in other directions.

To integrate the advantages of different architectures and address more complex engineering requirements, hybrid designs combining multiple TPMS structures have been proposed. Compared to uniform structures, hybrid TPMS structures demonstrate higher energy absorption under static loading. They also exhibit a more uniform stress distribution and more coordinated deformation. Furthermore, these hybrid designs help deflect crack propagation and delay structural failure. For instance, AlQaydi et al.^36^ fabricated a hybrid structure combining Primitive and IWP units via Laser Powder Bed Fusion (PBF-LB/M). Its mechanical performance was estimated using numerical simulations and quasi-static compression tests coupled with Digital Image Correlation. The results showed that the hybrid structure’s properties were intermediate between the two parent structures, enabling the design of lattices with tailored directional properties by adjusting the composition ratio. In another study, Zhang et al.^37^ investigated the effects of composition, transition layer width, relative density, and fusion mode on multi-stage energy absorption. Arranging TPMS structures sequentially along the loading direction in order of their yield strengths has been identified as the most effective approach to achieve this effect.

Despite these advances, existing research on linearly hybridized TPMS structures exhibits notable limitations. Most studies focus on enhancing overall mechanical properties under unidirectional loading applied along the hybridization direction. They frequently overlook the performance uncertainties in other loading directions. This narrow focus differs significantly from the complex loading conditions encountered in actual engineering applications. To address these complex requirements, multidimensional hybrid designs offer greater potential for tailoring mechanical performance. These advanced designs enable the simultaneous optimization of stiffness, strength, and energy absorption^38,39^. To explore this potential, this study proposes an in-plane hybrid design concept that effectively improves the energy absorption capacity of TPMS structures under compressive loading in different directions. A Sigmoid function was utilized to achieve a seamless integration of the Gyroid and IWP unit cells, resulting in the development of two novel in-plane hybrid structures, G-IWP-1 and G-IWP-2. These structures and their uniform counterparts were fabricated using Laser Powder Bed Fusion (PBF-LB/M). Their quasi-static compression behavior and energy absorption performance were systematically evaluated through both experimental tests and finite element simulations. The evaluation results highlight the advantages of combining multiple TPMS architectures into a single structure, providing a practical framework for designing porous lattices with superior mechanical efficiency. Ultimately, this study contributes to the advancement of multi-dimensional TPMS designs and offers a promising pathway for their application in lightweighting and energy-absorbing components.

## Materials and methods

For this study, two lattice topologies, Gyroid and IWP structures with specimen size of 24.46 mm, and in-plane hybrid structures of these two lattices are designed. All these lattice topologies are fabricated with Ti-6Al-4 V spherical powder by metal powder bed fusion, and structural characteristics of specimens are evaluated.

### Uniform structure

TPMS structures are generally described with implicit equations, which enables efficient parametric modelling. In order to define implicit surfaces, the trigonometric superposition function can be used. The general form of the function can be expressed as follows:

1 $$\varphi ({\mathbf{r}})=\sum\limits_{{k=1}}^{N} {{A_k}\cos \left[ {\frac{{2\pi ({{\mathbf{h}}_{\mathbf{k}}} \cdot {\mathbf{r}})}}{{{\lambda _k}}}+{P_k}} \right]} =C$$

where **r** is the spatial coordinate vector (*x*,* y*,* z*) in three dimensions. Here ${A_k}$, ${{\mathbf{h}}_{\mathbf{k}}}$, ${\lambda _k}$, ${P_k}$ represent the amplitude, the periodic vector, the periodic wavelength and the spatial phase shift respectively. The parameter *C* determines the volume ratio by specifying the geometry, thickness, and the structural properties.

In this study, the defining minimal surface equations of Gyroid^40^ and IWP can be expressed as follows:

Gyroid surfaces:

2 $${\varphi _G}=\sin \left( X \right)\cos \left( Y \right)+\sin \left( Y \right)\cos \left( Z \right)+\sin (Z)\cos \left( X \right)=C$$

IWP surfaces:

3 $$\begin{gathered} {\varphi _{IWP}}{\text{ = 2[}}\cos (X)\cos (Y)+\cos (Y)\cos (Z)+\cos (Z)\cos (X)] \\ - [\cos (2X)+\cos (2Y){\mathrm{+}}\cos (2Z)]\;{\text{= }}C \\ \end{gathered}$$

Where *X* = 2π*x*, *Y* = 2π*y*, *Z* = 2π*z*, *x*∈R, *y*∈R, *z*∈R. Curvature parameter *C* determines the position of the isosurface in three-dimensional space, therefore, controls the relative density of the TPMS structure. When *C* = 0, these equations describe isosurfaces with zero-level symmetry, which represent the standard form shell-based Gyroid and IWP structure.

Previous studies recommend an arrangement of at least 4 × 4 × 4 unit cells to ensure the measured mechanical properties are representative^41^. Numerical simulations have confirmed that a lattice of this size provides stiffness comparable to the asymptotic modulus of an infinite array, thereby minimizing boundary effects^42^. Therefore, using Eq. (2) and Eqs. (3), 4 × 4 × 4 lattice arrangements of the uniform Gyroid and IWP structures are generated.

### Design strategy of in-plane hybridization

In this study, Gyroid and Schoen IWP unit cells were selected to construct the hybrid structures due to their large specific surface areas. To seamlessly integrate the two topologies, a connection function was employed. This strategy ensures geometric continuity and avoids abrupt transitions that could compromise structural integrity. The in-plane hybridization was implemented along both the x and y directions, as described by the following connection functions:

In x direction:

4 $$\begin{gathered} {\gamma _{\mathrm{n}}}=(1 - {A_1})({\varphi _1} - {\varphi _2})+(1 - {A_2})({\varphi _2} - {\varphi _3})+(1 - {A_3})({\varphi _3} - {\varphi _4}) \\ + \cdots +(1 - {A_{m - 2}})({\varphi _{m - 2}} - {\varphi _{m - 1}})+(1 - {A_{m - 1}}){\varphi _{m - 1}}+{A_{m - 1}}{\varphi _m} \\ \end{gathered}$$

And in y direction:

5 $$f={\gamma _1}{B_1}+{\gamma _2}{B_1}{B_2}+{\gamma _3}{B_2}{B_3}+ \cdots +{\gamma _{m - 1}}{B_{m - 2}}{B_{m - 1}}+{\gamma _m}{B_{m - 1}}$$

where${A_m}$ and ${B_m}$ are sigmoid functions that help prevent abrupt changes in the transition region and define the blending ratio between the two structures. The sigmoid function can be expressed as:

6 $$\alpha (x,y,z)=\frac{1}{{1+{e^{ - k\eta (x,y,z)}}}}$$

**Fig. 1:**
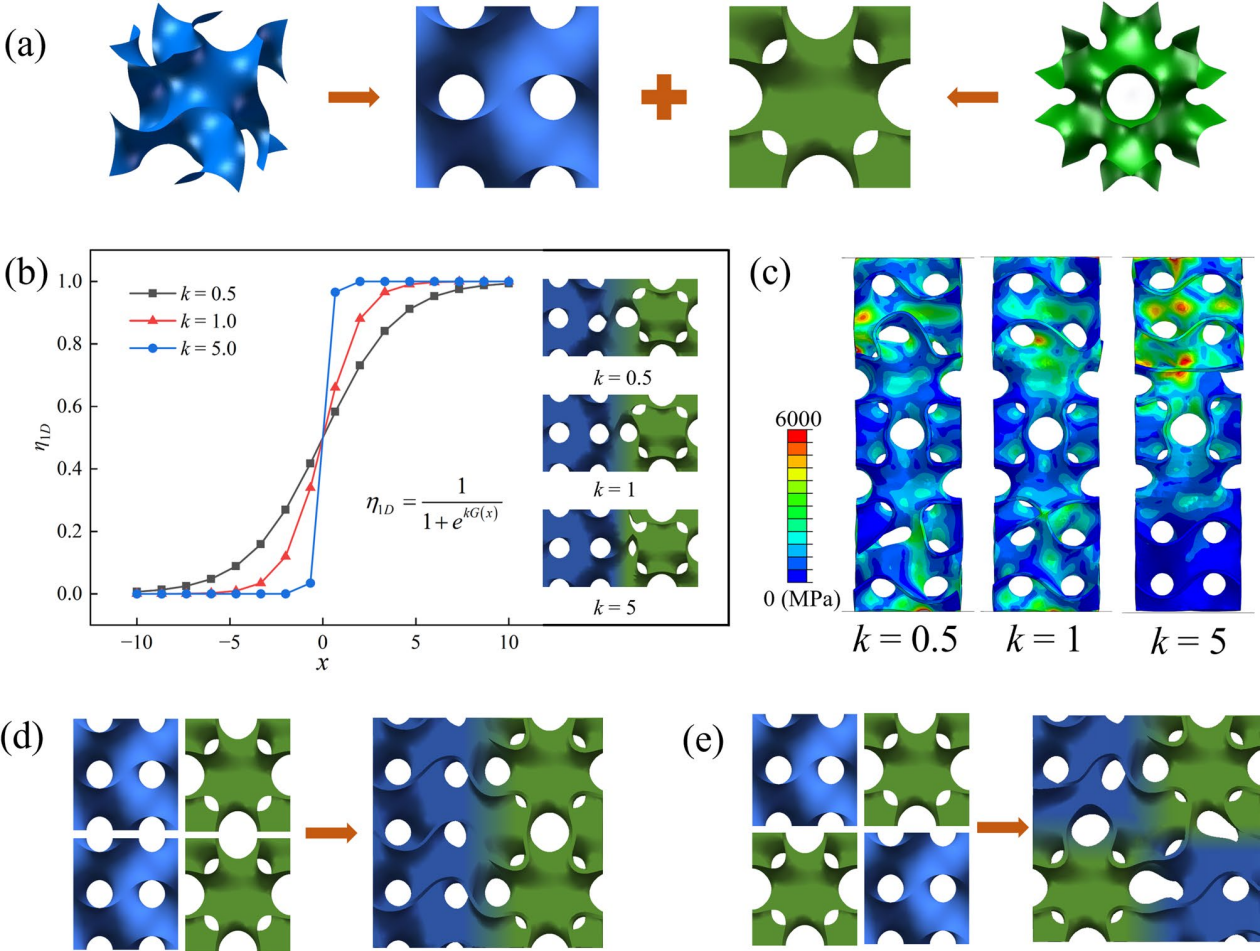
In-plane hybridization design: (**a**) Gyroid and IWP cell; (**b**) different *k* values; (**c**) Effect of k value in sigmoid function on mechanical properties of hybrid region; (**d**) G-IWP-1; (**e**) G-IWP-2.

The range of $\alpha (x,y,z)$ is from 0 to 1, where the function $\eta (x,y,z)=0$determines the midpoint of the transition between the two structures, and the parameter *k* controls the steepness of this transition. A smaller *k* value results in a more gradual sigmoid curve, while larger values lead to a steeper transition, potentially causing abrupt changes in mechanical or geometric properties. Figure 1(b) showcases the influence of different *k* values (*k* = 0.5, *k* = 1 and *k* = 5) on the fusion zone, a *k* value of 1 yields the most visually smooth and uniform transition interface. To further provide a mechanical justification for this geometric selection, the stress distribution within the hybrid regions under compression was evaluated for the different *k* values, as illustrated in Fig. 1(c). The results demonstrate that a steep topological transition (*k* = 5) induces the most severe stress concentration at the fusion boundary. Conversely, an overly gradual transition (*k* = 0.5) distorts the parent unit cells over a broader area, resulting in a more uneven stress distribution across the hybrid region. The *k* = 1 configuration demonstrates the optimal balance, effectively minimizing interfacial stress concentrations while maintaining a highly uniform stress state and the structural integrity of both the Gyroid and IWP topologies. This choice is consistent with previous studies that have successfully employed a *k*-value of 1^25,26,37^. Therefore, considering the need to achieve a gradual transition area while preserving the unique characteristics of each individual structure, the *k* parameter was set to 1 for this study.

In this study, Gyroid lattice and IWP lattice are hybridized in-plane in two forms. The first hybrid form is Gyroid and IWP unit cells are arranged side by side, and repeated in each layer as shown in Fig. 1(d); the second form is Gyroid and IWP unit cells are arranged side by side, the arrangement order is reversed in next layer, as shown in Fig. 1(e). Previous studies, including the systematic evaluations from our previous work^39^, have established that the mechanical properties of finite periodic structures rapidly converge, and boundary size effects significantly diminish with an increasing number of unit cells^26,43^. Consequently, an array of sufficient dimensions can effectively eliminate size effects and accurately represent the macroscopic bulk properties of the structure. To ensure consistency with the uniform structures and based on the rationale previously discussed, both hybrid designs were then constructed as 4 × 4 × 4 lattice arrangements.

### Base material

The base material used in this study is Ti-6Al-4 V alloy, and the particle size and distribution characteristics of the titanium alloy were characterized using a Malvern Mastersizer 2000 laser particle size analyzer.

**Fig. 2:**
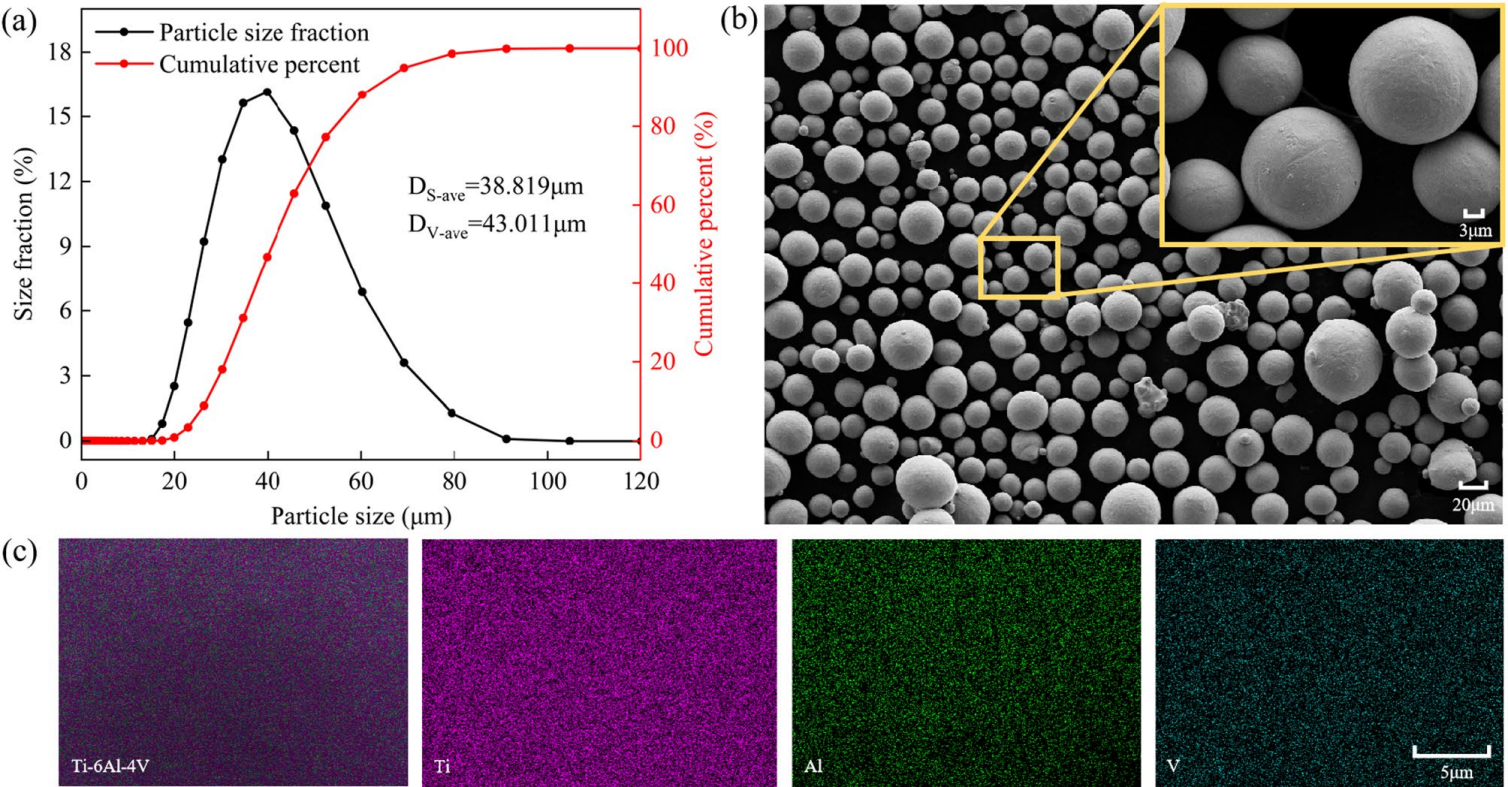
(**a**) Particle size distribution of titanium alloy powder; (**b**) SEM of titanium alloy powder; (**c**) Elemental distribution of the titanium alloy powder.

Based on the analytical results, the particle size ranges from 17.378 μm to 104.713 μm with average diameter 43.011 μm as shown in Fig. 2(a) below. Specifically, the particle size under 20 μm and over 70 μm is less than 5%. The characteristic values of the particle size distribution, namely D_0.1_, D_0.5_ and D_0.9_, are respectively 26.857 μm, 40.918 μm and 62.142 μm. This distribution characteristic indicates that the powder possesses an appropriate particle size uniformity, which meets the technical requirements of the selective laser melting (PBF-LB/M) process for powder flowability and spreading uniformity.

To observe the microstructural morphology of the titanium alloy powder, scanning electron microscopy (SEM, Zeiss Ultra Plus) was utilized. As shown in Fig. 2(b), the powder particles exhibit a spherical shape with smooth surfaces, ensuring uniform spreading and melt pool wettability. No satellite particles were observed.

The elemental distribution of the titanium alloy powder was analyzed using a SEM equipped with energy-dispersive X-ray spectroscopy (EDS) under an accelerating voltage of 25 kV. As illustrated in Fig. 2(c), the constituent elements Ti, Al, and V exhibit uniform distribution without macroscopic segregation. The powder’s favorable characteristics, including appropriate particle size distribution and smooth surface morphology, ensure excellent flowability. This property is critical for achieving uniform powder spreading during additive manufacturing processes. These inherent qualities make the Ti-6Al-4 V powder ideal for fabricating various TPMS structures via the EOS M290 3D printer using the powder bed fusion with laser beam melting (PBF-LB/M) method. Chemical composition analysis (ICP-AES, iCAP 6300) confirmed a composition of 90.55 wt% Ti, 5.39 wt% Al, and 4.05 wt% V.

### Fabrication and characterization

All Triply Periodic Minimal Surface (TPMS) structures specimens discussed in this paper were fabricated using Laser Powder Bed Fusion (PBF-LB/M). The uniform structures include Gyroid and IWP, while the hybrid structures are designated as G-IWP-1 and G-IWP-2. Table 1 presents the design specifications and key characteristics for each type of TPMS structure specimens.

**Table 1:** Design and characteristics of TPMS structure specimens.

Type	Lattice arrangement	Specimen edge length (mm)	Theoretical relative density (%)	Measured relative density (%)	Mass (g)
Gyroid	4 × 4 × 4	24.46	18.66	22.07	13.54
IWP	4 × 4 × 4	24.46	22.06	26.83	16.53
G-IWP-1	4 × 4 × 4	24.46	20.66	24.76	15.45
G-IWP-2	4 × 4 × 4	24.46	20.64	24.64	15.15

**Fig. 3:**
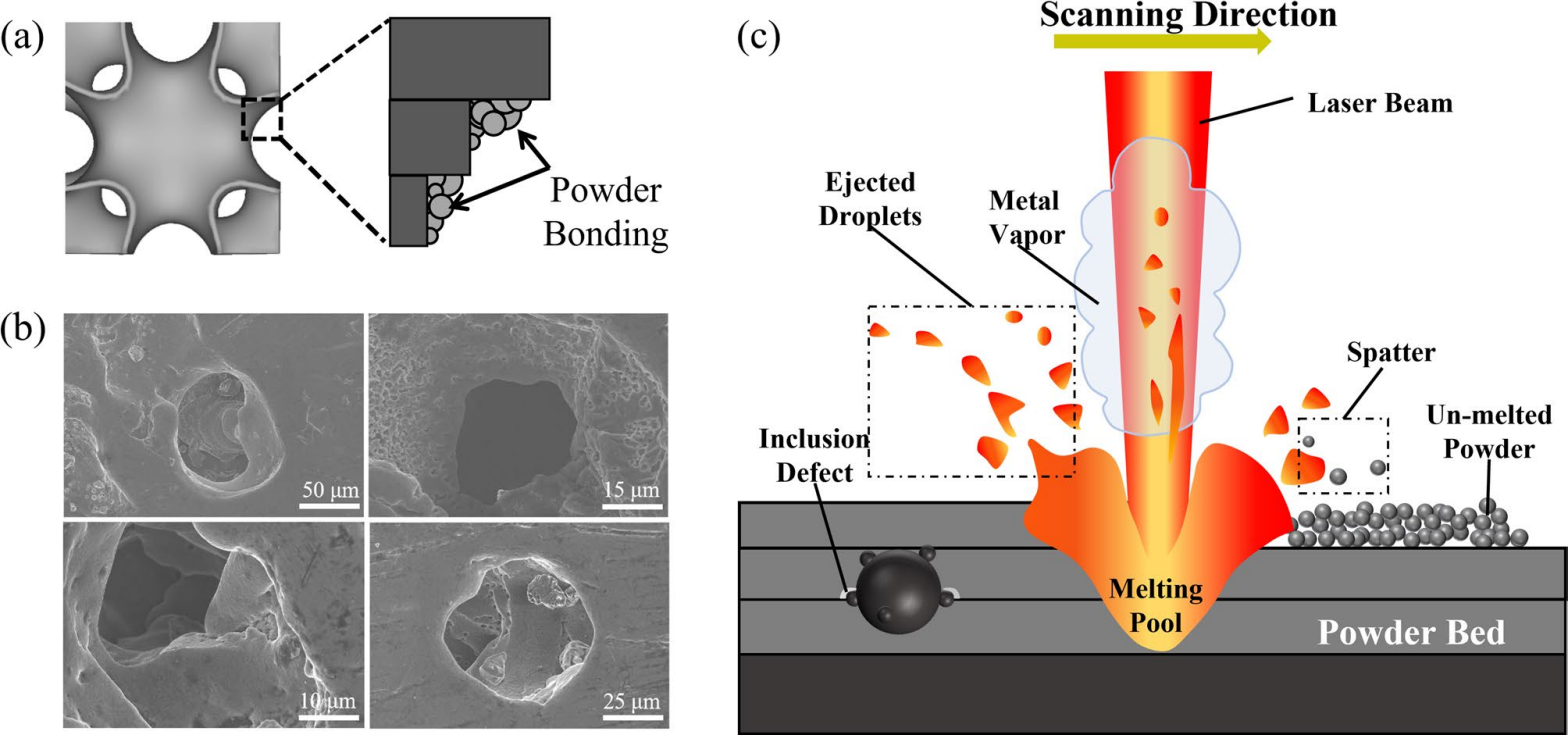
(**a**) Powder bonding at layer edges in PBF-LB/M-fabricated TPMS structures. (**b**) Porosity and unmelted powder in structure interiors. (**c**) The splash phenomenon and the principle of internal hole generation.

The deviation between the measured and theoretical relative densities primarily stems from manufacturing imperfections inherent in the PBF-LB/M process. As shown in Fig. 3(a), while TPMS structures are ideally designed with smooth and continuous surfaces, the actual surfaces produced by PBF-LB/M are characterized by a stepped, layered appearance. At the junctions between these layers, residual unmelted powder particles tend to accumulate. These fundamental process limitations inevitably lead to various defects within the fabricated TPMS structure. Mechanically, while the adhered unmelted powder increases the apparent mass and measured relative density, its contribution to the structural load-bearing capacity is limited. Furthermore, the stepped surface topology can locally reduce the effective load-bearing cross-sectional area of the struts, which partially accounts for the slight differences in initial structural stiffness and yield strength compared to the theoretical models.

Beyond these density and surface characteristics, an examination of the internal microstructure reveals defects inherent to PBF-LB/M-fabricated TPMS structures. The structural specimens were sectioned using wire-electro discharge machining and subsequently analyzed via SEM. As illustrated in Fig. 3(b), the internal porosity exhibited irregular morphologies and variable sizes. Notably, some pores contained partially unmelted powder particles adhering to their internal surfaces. These process-induced defects are primarily attributed to spattering during the PBF-LB/M process, a phenomenon that encompasses both the ejection of molten droplets and the dispersion of surrounding powder particles.

There are two main physical processes involved in pore formation. First, vaporization-induced depression of the melt pool creates recoil pressure, which then causes molten metal spatter and the formation of voids. Second, the Marangoni effect, driven by surface tension gradients, leads to a convective flow of molten metal from hotter to cooler areas within the melt pool. If this flow velocity exceeds certain limits, it results in secondary liquid metal spatter. These ejected droplets hit surrounding unmelted powder particles, causing a chain reaction of powder dispersion. This sequential spatter mechanism ultimately degrades the uniformity of the powder bed during later deposition cycles, leading to the internal porosity we observe. From a solid mechanics perspective, these randomly distributed internal pores can act as localized stress concentrators during quasi-static compression. While they may influence the threshold for micro-crack initiation within certain struts, the macroscopic energy absorption mechanisms of the structure remain dominant. Nevertheless, these localized yielding phenomena can introduce minor fluctuations in the plateau stress and partially explain the deviations in the specific energy absorption (SEA) capacity observed between the actual experiments and the idealized simulations.

### Quasi-static compression

**Fig. 4:**
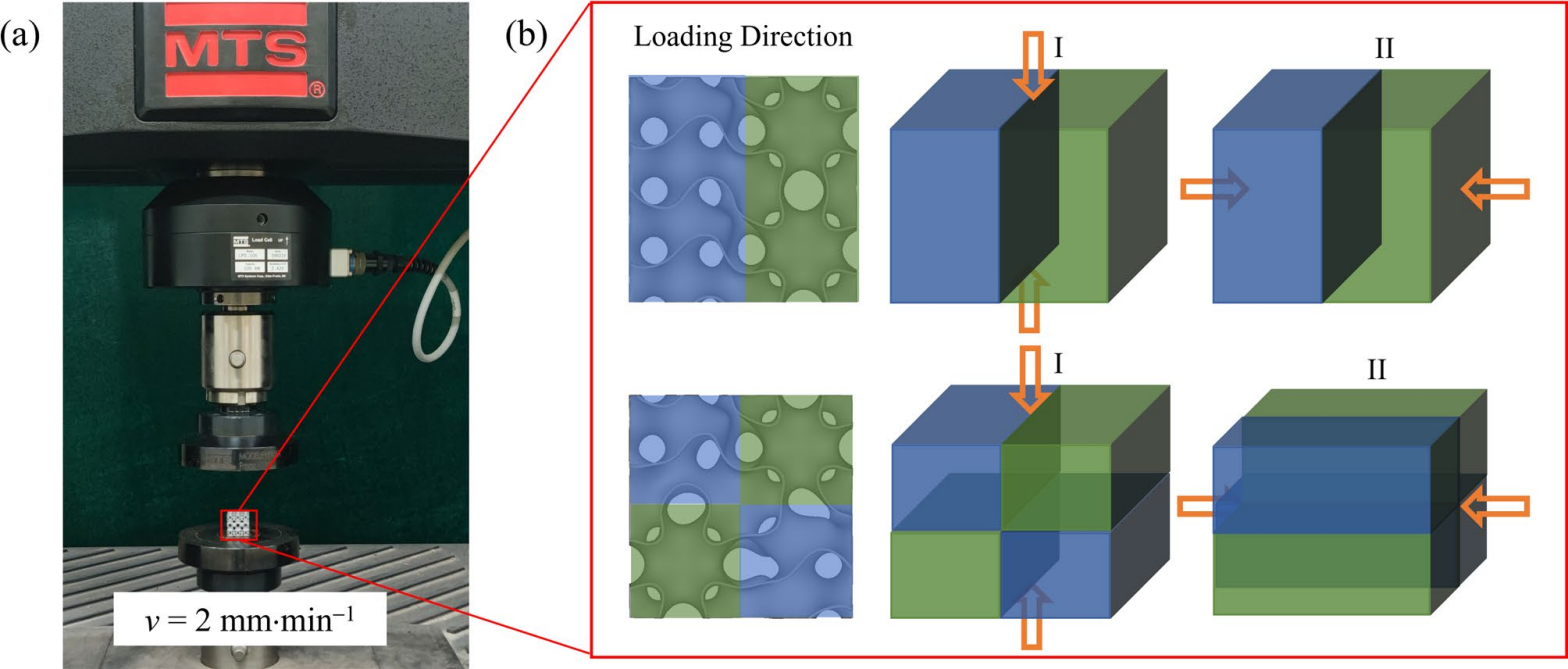
(**a**) Quasi-static compression test setup; (**b**) loading direction diagram.

The uniaxial compressive behavior of both the uniform and hybrid TPMS structures was evaluated using an MTS322 hydraulic testing machine, as depicted in the experimental setup in Fig. 4(a). To approximate quasi-static conditions, all tests were conducted under displacement control at a constant crosshead speed of 2 mm/min. Throughout each test, load-displacement data were acquired at a sampling frequency of 10 Hz. To ensure a uniaxial stress state, specimens were carefully centered on the compression platens. For the hybrid TPMS structures, the influence of loading anisotropy was investigated by applying compression along two distinct directions I and II, as illustrated in Fig. 4(b). To ensure the repeatability of the experimental results and minimize random errors, three identical specimens were fabricated and tested for each structural configuration. The median stress-strain curve from the three replicate tests was selected as the representative result for subsequent analysis^37,44,45^.

**Fig. 5:**
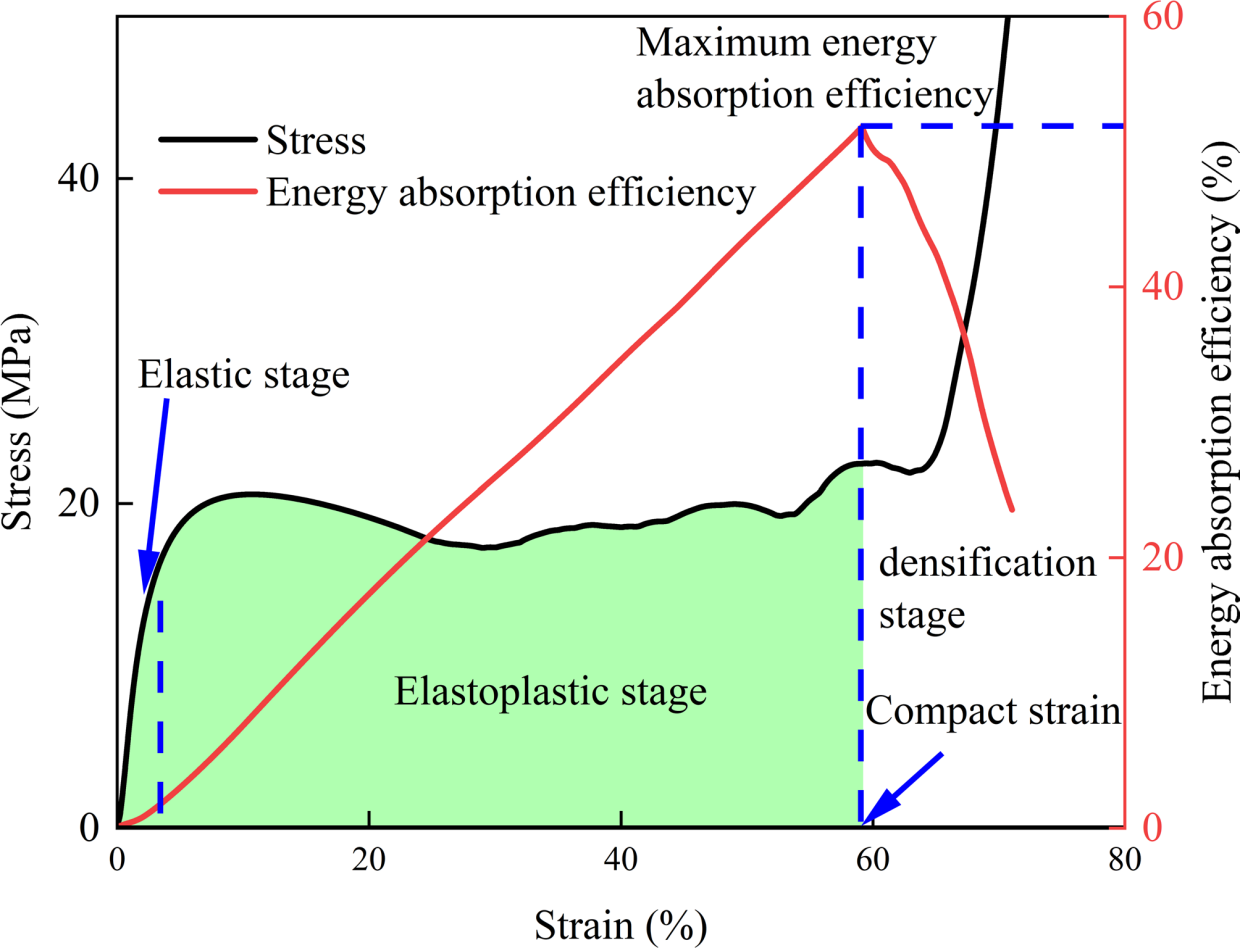
Schematic diagram for obtaining compression performance index based on stress-strain curve.

The compressive behavior of the TPMS structures was evaluated using several key indicators: Young’s modulus (*E*), densification strain (*ε*_d_), plateau stress (*σ*_pl_), and specific energy absorption (*SEA*). Figure 5 provides a schematic illustration of how these metrics are determined from the characteristic stress-strain curve of a cellular solid.

The effective Young’s modulus (*E*) is determined from the slope of the linear-elastic portion of the stress-strain curve.

For a porous structure under compression, the energy absorption efficiency is calculated from the stress-strain curve at a given strain *ε*:

7 $$\eta (\varepsilon )=\frac{1}{{\sigma (\varepsilon )}}\int_{0}^{\varepsilon } {\sigma (\varepsilon )d\varepsilon }$$

The densification strain (*ε*_d_) is identified as the strain at which the efficiency function in Eq. (7), reaches its maximum value as:

8 $${\left. {\frac{{d\eta (\varepsilon )}}{{d\varepsilon }}} \right|_\varepsilon }_{{={\varepsilon _d}}}=0$$

The yield strength (plateau stress, *σ*_pl_) represents the average load-bearing capacity of the porous structure during progressive collapse. It is defined as the mean stress within the strain interval from 0 to *ε*_d_, which can be expressed as:

9 $${\sigma _{pl}}=\frac{{\int_{{\mathrm{0}}}^{{{\varepsilon _{\mathrm{d}}}}} {\sigma (\varepsilon )} d\varepsilon }}{{{\varepsilon _{\mathrm{d}}}}}$$

The specific energy absorption (*SEA*) is a key indicator of a structure’s energy dissipation capability. It is defined as the total energy absorbed per unit mass up to the onset of densification:

10 $$SEA=\frac{{\int_{0}^{l} {F(x)dx} }}{m}=\frac{{V\int_{0}^{{{\varepsilon _d}}} {\sigma (\varepsilon )d\varepsilon } }}{m}$$

where *F(x)* is the axial compressive force, *l* is the displacement corresponding to the densification strain. *V* is the volume of a cube with the same length, width, and height as the TPMS structure, and *m* denotes the mass of the corresponding structure.

With these equations, the key mechanical performance indicators for the uniform and hybrid TPMS architectures were calculated from the quasi-static compression data.

### Numerical simulation

To further understand the mechanical response and deformation mechanisms, finite element (FE) simulations were conducted using the commercial software Abaqus/Explicit. The geometric models were generated based on the implicit functions described in Sect.  2.1 and 2.2. These models represent idealized structures and do not account for manufacturing defects such as surface roughness or internal porosity. The structures were discretized using six-node linear triangular prism elements (C3D6) with a uniform wall thickness of 0.4 mm. A mesh convergence study determined an average element size of 0.3 mm to ensure calculation accuracy and efficiency.

The boundary conditions were defined to replicate the quasi-static compression experiments. The TPMS model was positioned between two rigid plates. All degrees of freedom of the bottom plate were fixed. A vertical displacement was applied to the top plate at a constant velocity of 1 mm/min, which ensured that the simulation remained under quasi-static conditions. The lateral movement and rotation of the top plate were restricted to ensure uniaxial compression. A general contact interaction was defined using “Hard” contact for normal behavior and a friction coefficient of 0.2 for tangential behavior.

The mechanical behavior of the Ti-6Al-4 V alloy was described using the Johnson-Cook (J-C) constitutive model. This model incorporates strain hardening, strain rate effects, and thermal softening. The flow stress is expressed as follows:

11 $$\sigma =\left( {A+B{\varepsilon ^n}} \right)\left( {1+C\ln \frac{{\dot {\varepsilon }}}{{{{\dot {\varepsilon }}_0}}}} \right)\left( {1 - {{\left[ {\frac{{T - {T_{room}}}}{{{T_m} - {T_{room}}}}} \right]}^m}} \right)$$

where *A* is the initial yield stress, *B* is the hardening coefficient, and *C* is the strain rate coefficient. The variable *n* is the strain hardening exponent, and *m* is the thermal softening exponent. $\varepsilon$ represents the effective plastic strain, and $\frac{{\dot {\varepsilon }}}{{{{\dot {\varepsilon }}_0}}}$ is the normalized equivalent plastic strain rate. The variables *T*,${T_m}$, and ${T_{room}}$denote the material temperature, melting temperature, and room temperature, respectively. To predict material failure, a J-C damage model was employed. The damage initiation strain is defined by:

12 $${\varepsilon _D}=\left[ {{d_1}+{d_2}\exp ( - {d_3}\eta )} \right]\left[ {1+{d_4}\ln \frac{{\dot {\varepsilon }}}{{{{\dot {\varepsilon }}_0}}}} \right]\left( {1+{d_5}\left[ {\frac{{T - {T_{room}}}}{{{T_m} - {T_{room}}}}} \right]} \right)$$

where ${d_1}$, ${d_2}$, and ${d_3}$ are parameters related to stress triaxiality. The parameters ${d_4}$ and ${d_5}$ are associated with the strain rate and temperature, respectively. The variable $\eta$ represents the stress triaxiality, which is defined as $\eta =p/\overline {\sigma }$. In this equation, *p* is the mean stress and $\overline {\sigma }$ is the von Mises equivalent stress.

The specific material parameters used in Eq. (11) and Eq. (12) were adopted from the work of G. Kay^46^, Zhou et al.^47^ and Cansizoglu et al.^48^. These parameters are summarized in Tables 2 and 3.

**Table 2:** J-C constitutive model parameters of Ti-6Al-4 V alloy.

Parameter	ρ (g/cm^3^)	E (GPa)	ν	A (MPa)	B (MPa)	*n*	C	q
Value	4.43	110	0.3	1098	1092	0.93	0.014	1.1

**Table 3:** J-C damage model parameters of Ti-6Al-4 V alloy.

Parameter	d_1_	d_2_	d_3_	d_4_	d_5_	G_f_ (kJ/m^2^)
Value	-0.09	0.27	0.48	0.014	3.87	86

## Results and discussion

### The uniform structures

**Fig. 6:**
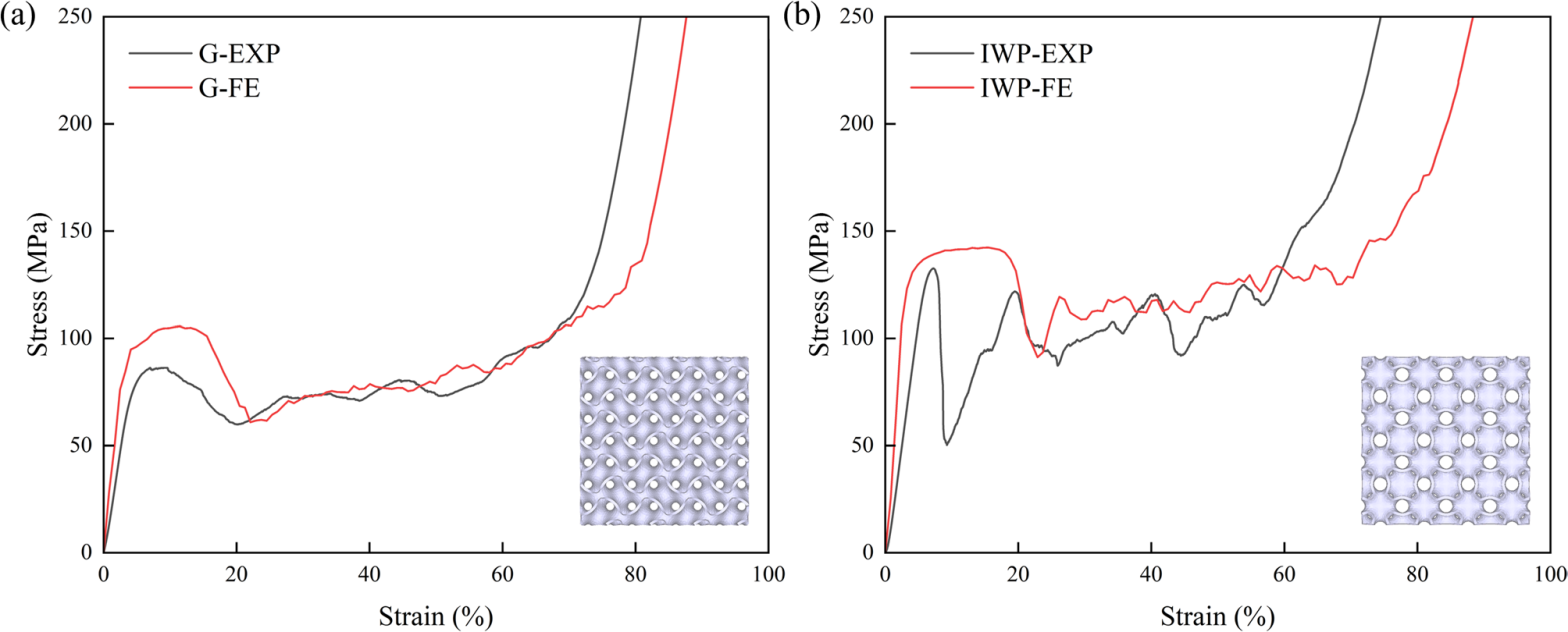
Experimental and FE simulation stress-strain curves of uniform structures under quasi-static compression: (**a**) Gyroid; (**b**) IWP.

**Fig. 7:**
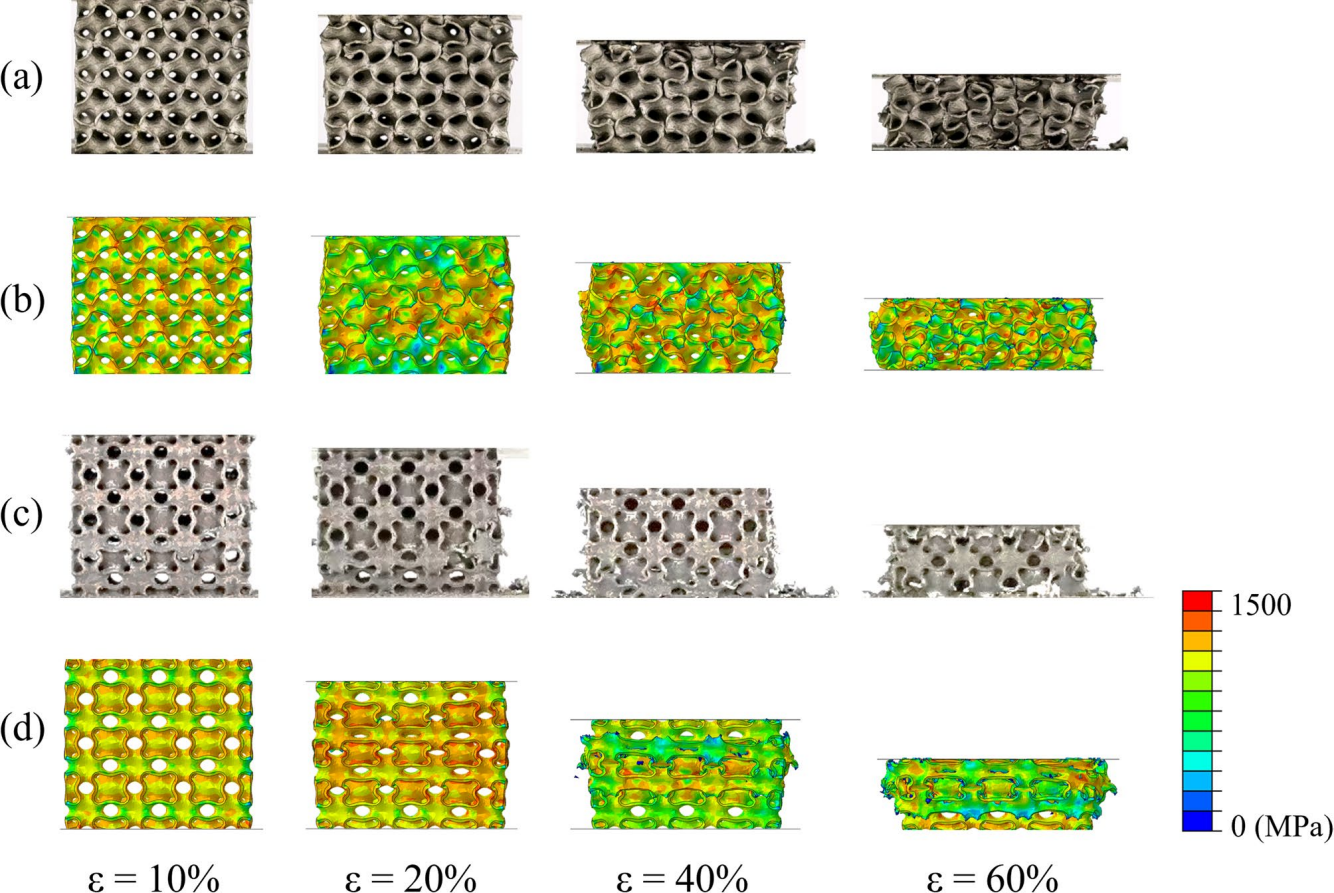
Comparison of deformation modes between experimental results and FE simulations for uniform structures at progressive strains: (**a**) Gyroid (Experiment); (**b**) Gyroid (FE); (**c**) IWP (Experiment); (**d**) IWP (FE).

To compare their mechanical performance, uniform Gyroid and IWP structures were subjected to quasi-static compression. The resulting stress-strain responses and corresponding deformation modes are presented in Figs. 6 and 7, respectively.

During the plateau phase, which involves progressive and layer-by-layer collapse, significant differences in the failure behavior of the two structures emerge. The IWP structure attains a higher peak stress than the Gyroid structure, indicating superior initial load-bearing capacity. Following this peak, however, the response of the two architectures diverges notably. The Gyroid structure’s plateau is smoother and exhibits smaller-amplitude fluctuations, whereas the IWP curve is characterized by larger, more abrupt stress drops and rises. This suggests that the Gyroid structure undergoes a more stable and progressive collapse.

Consistent with the visual evidence of progressive collapse shown in Fig. 7, this interpretation is supported by quantitative analysis of the failure events. The IWP structure displays a more sudden, brittle-like failure of individual layers, reaching its first significant stress minimum at approximately 10% strain. In contrast, the Gyroid structure reaches its lowest stress point much later, at roughly 20% strain. Furthermore, the magnitude of the stress drop is more severe for the IWP, with the first valley falling to just 38.06% of its initial peak stress, compared to 59.62% for the Gyroid structure. This overall brittle failure mechanism, governed by the fracture of sheet surfaces, is consistent with previous findings for additively manufactured metallic lattices reported by Sun et al.^49^, Weißmann et al.^50^ and Van Hooreweder et al.^51^.

### In-plane hybrid structures

**Fig. 8:**
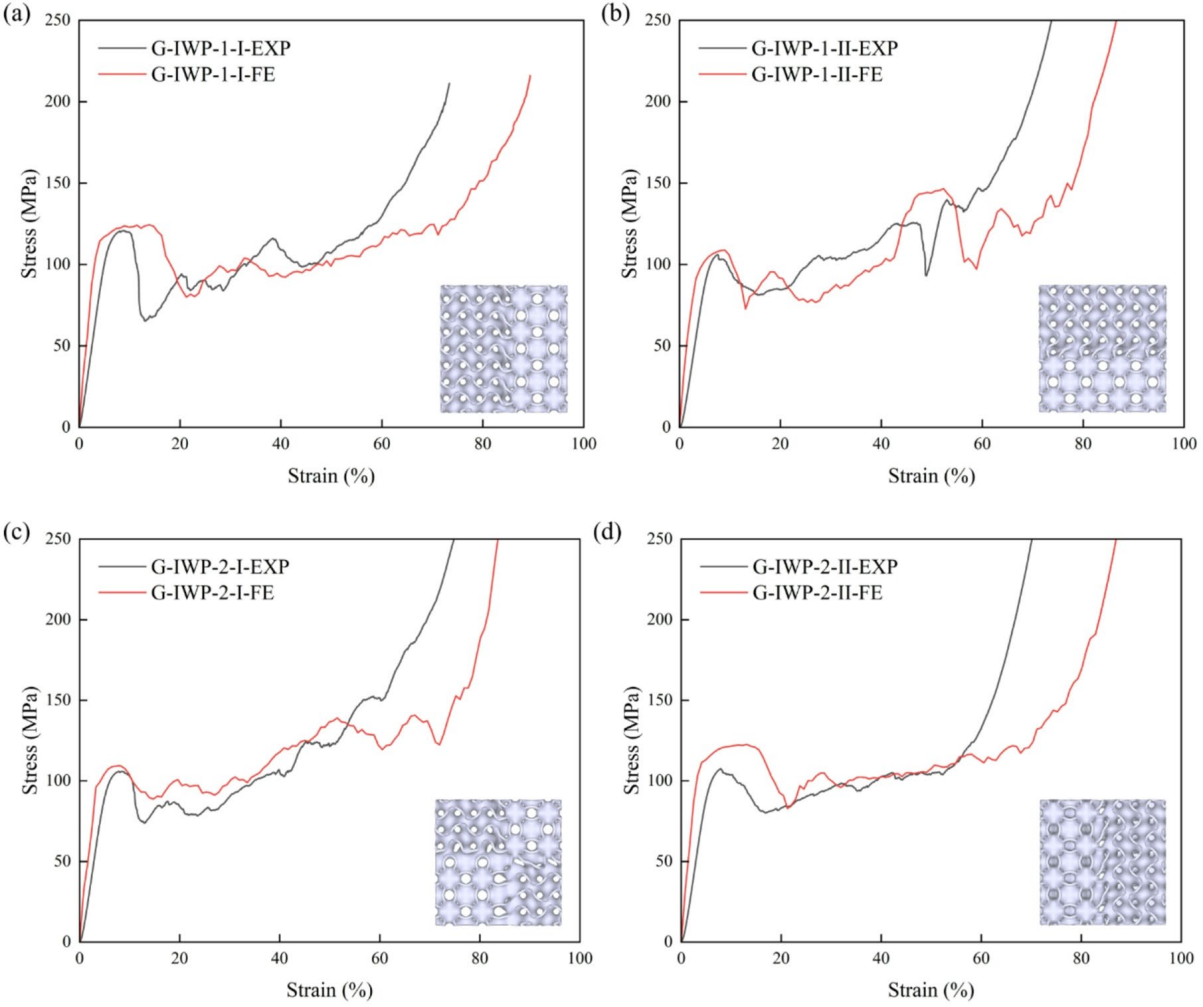
Experimental and FE simulation stress-strain curves of hybrid structures under quasi-static compression: (**a**) G-IWP-1-I; (**b**) G-IWP-1-II; (**c**) G-IWP-2-I; (**d**) G-IWP-2-II.

Quasi-static compression tests were conducted on the G-IWP-1 and G-IWP-2 hybrid structures along two principal directions (I and II). The results reveal significant mechanical anisotropy. Figure 8 details the stress-strain responses and corresponding deformation sequences for each test.

As shown in Fig. 8, a good correspondence is observed between the experimental results and FE predictions. For the G-IWP-1 structure, discrepancies in the plateau region are attributed to the idealized nature of the FE models. The stress-strain curve for the case of loading direction I, presented in Fig. 8(a), shows a sharp stress drop after yielding, which reflects the sudden, localized failure observed in the sample. In contrast, the FE model does not include material defects, so it is expected to predict a higher peak strength and a smoother collapse response. Compared with direction I, the direction II case in Fig. 8(b) shows a closer agreement between experiment and FE simulation, where the FE simulation successfully captures the overall yield behavior and provides a good average representation of the complex stress oscillations seen in the experiment.

For G-IWP-2, the FE simulation demonstrates good predictive accuracy in both loading directions. The model not only matches the initial elastic stiffness but also successfully captures the complex post-yield behavior. This includes the initial stress peak, the subsequent strain-softening drop, and the overall shape and magnitude of the plateau region. In particular, the simulation accurately reproduces the rapid stress drop associated with shear failure in direction I case (Fig. 8(c)) and the more gradual plateau characteristic of the layer-by-layer collapse in direction II case (Fig. 8(d)). The numerical curves closely track the experimental trends and provide a good representation of the mechanical response.

**Fig. 9:**
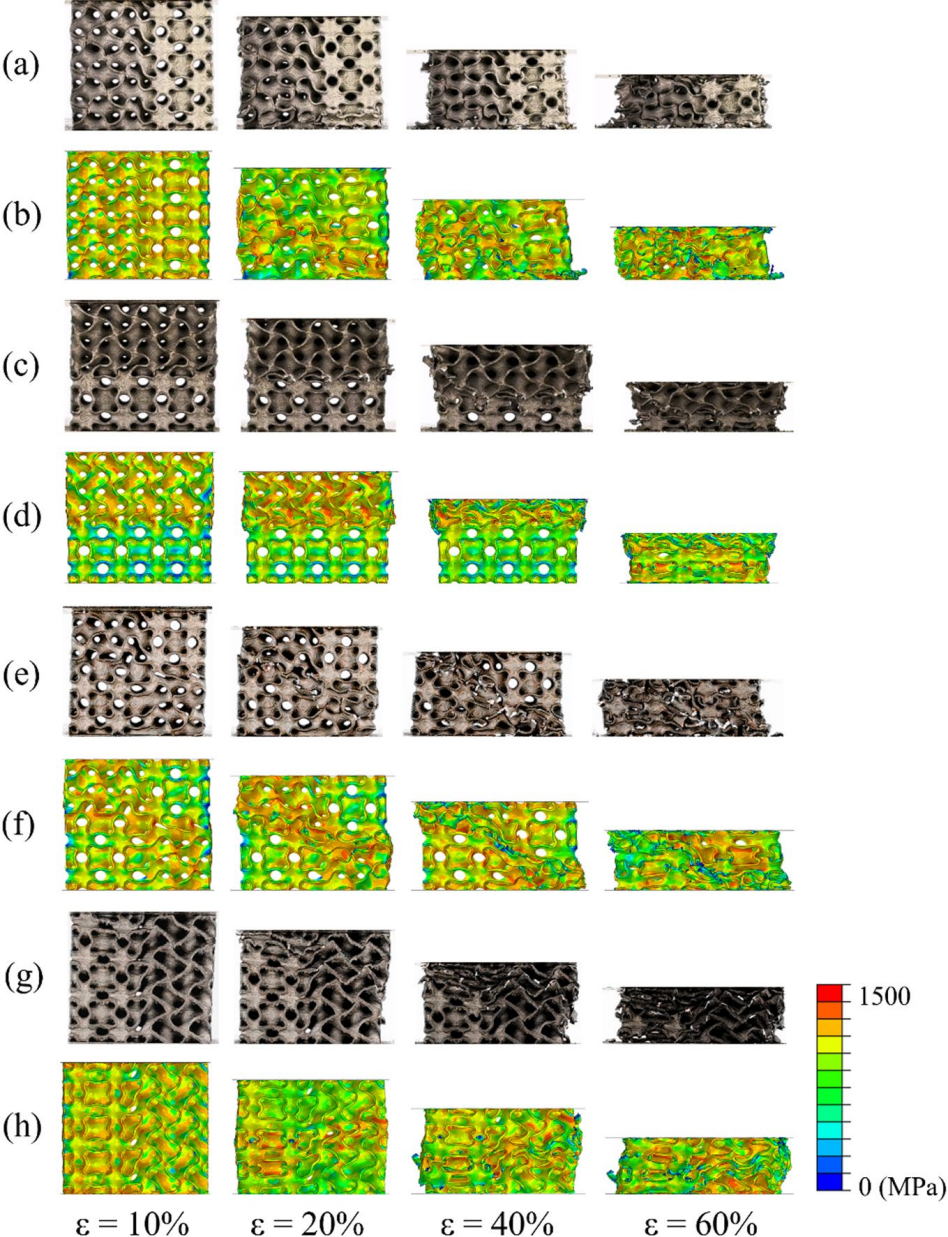
Comparison of deformation modes between experimental results and FE simulations for hybrid structures at progressive strains: (**a**) G-IWP-1-I (Experiment); (**b**) G-IWP-1-I (FE); (**c**) G-IWP-1-II (Experiment); (**d**) G-IWP-1-II (FE); (**e**) G-IWP-2-I (Experiment); (**f**) G-IWP-2-I (FE); (**g**) G-IWP-2-II (Experiment); (**h**) G-IWP-2-II (FE).

For the G-IWP-1 structure, the deformation comparisons reveal how the loading direction profoundly influences its failure mechanism. Under compression in Direction I, the hybrid structure is effectively divided into a strong yield strength region (IWP) and a weak yield strength region (Gyroid) parallel to the loading axis. While this parallel configuration allows the IWP domains to provide robust initial structural support, the stark contrast in local stiffness causes the experimental specimen to exhibit a less stable collapse. Failure initiates through localized fracture at the bottom layer and then propagates upward, as shown in Fig. 9(a). In contrast, the FE simulation in Fig. 9(b) exhibits a more distributed plastic deformation due to its idealized geometry. This difference in deformation behavior explains the pronounced fluctuations in the experimental stress plateau compared with the smoother numerical response. In Direction II, loading as shown in Fig. 9(c) and (d) produces a highly stable, progressive failure in both the experiment and the simulation. In this orientation, the structure yields sequentially, strictly following the gradient of yield strengths across the unit cell layers. Deformation begins in the compliant Gyroid region and then proceeds sequentially through the structure, creating a multi-stage plateau effect which explains the smoother stress plateau and the more controlled energy absorption.

The G-IWP-2 structure displays a different form of anisotropy, with the FE simulations showing good consistency with the experimental observations presented in Fig. 9(e)-(h). When compressed in direction I case, both the experiment and simulation show a distinct shear-dominated failure, with a shear band at approximately 45 degrees forming in the Gyroid region. Despite this localized shear failure, the specific spatial configuration creates a strong complementary effect. The integrated IWP structure acts as the primary load-bearing skeleton, providing directional strengthening along specific load paths. This synergistic support successfully mitigates the negative impacts of the Gyroid structure’s inherent tendency for widespread fracture. This mechanism corresponds to the rapid post-peak stress drop and early densification seen in the stress-strain curve. Conversely, compression in direction II case results in a conventional layer-by-layer collapse in both the experiment and simulation. Similar to G-IWP-1, loading in this direction leverages the gradient of yield strengths. Furthermore, the alternating checkerboard arrangement of strong and weak regions acts as a topological barrier that effectively interrupts continuous diagonal shear paths, suppressing the formation of macroscopic shear bands. This mode is characterized by a more gradual transition through the plateau region and delayed densification, suggesting a more progressive failure process. The close match in deformation topology between experiment and simulation confirms that the FE model correctly describes the internal stress redistribution and progressive failure process, despite minor discrepancies arising from the brittle fracture inherent to the manufacturing defects in the specimens.

In summary, the results highlight the tunable nature of hybrid TPMS designs. G-IWP-1 exhibits pronounced anisotropy in its primary mechanical properties, offering a clear trade-off between high strength (direction I case) and stable energy absorption (direction II case). The FE analysis confirmed this behavior, linking the instability in direction I case to high stress concentrations. G-IWP-2, while more isotropic regarding peak strength, demonstrates significant anisotropy in its failure mechanism—shear-band formation versus sequential crushing. This critical difference in failure mode was also accurately captured by the simulations. This allows its post-yield response and failure trajectory to be effectively tailored by the loading orientation. However, the simulations represent an idealized case. They show failure initiating at a later stage and mainly in the central high-stress regions. In contrast, the experiments exhibit earlier failure that is strongly influenced by local defects.

**Fig. 10:**
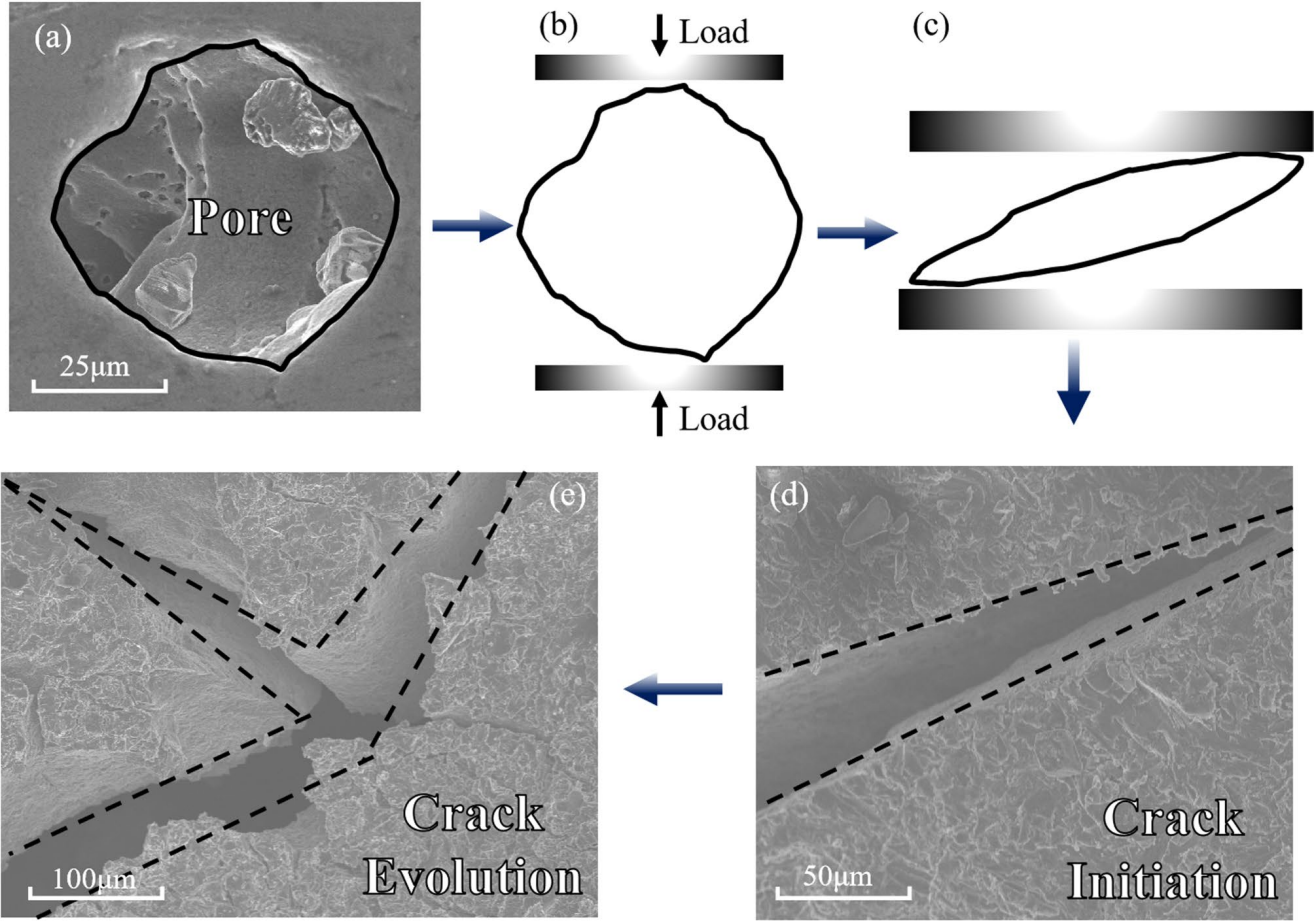
Sequence of crack formation of structures under quasi-static compression.

Fundamentally, the brittle failure mechanisms detailed above can be attributed to inherent microstructural defects common in additively manufactured components. Under quasi-static compression, these process-induced pores and voids act as stress concentrators. While the macroscopic load is compressive, these defects, particularly those with sharp corners, create a localized complex stress state. Although compression does not directly cause tearing, it induces lateral expansion in the surrounding material. This expansion, combined with the stress concentration at the defect tips, generates significant local tensile stresses. The material surrounding these defects deforms first, leading to crack initiation. As compression continues, these cracks propagate through the structure, culminating in the localized fractures and eventual failure modes observed experimentally. A schematic of this crack evolution mechanism is illustrated in Fig. 10. This reality-based behavior explains the frequent divergence from predictions made by idealized finite element models, which typically assume a perfect, defect-free geometry. Furthermore, other manufacturing limitations, such as partially melted powder adhesion and internal residual stresses, contribute to the discrepancies between the experimentally observed mechanical response.

### Mechanical properties

With the properties of the solid material defined, the focus now shifts to the mechanical performance of the TPMS structures under compression. A comprehensive summary of these properties is presented in Table 4.

**Table 4:** Mechanical Properties of Uniform and Hybrid TPMS Structures.

Gyroid	Direction	E (MPa)	ε_d_	σ_pl_ (MPa)	SEA (J/g)
		1896.80	0.570	71.65	44.13
IWP		1982.05	0.570	98.39	49.62
G-IWP-1	I	1966.24	0.589	95.81	53.44
G-IWP-1	II	1583.65	0.489	95.89	44.43
G-IWP-2	I	1693.33	0.609	99.90	58.78
G-IWP-2	II	1648.41	0.523	90.87	45.88

As indicated by the data in Table 4, structural design has a significant impact on the mechanical properties of the structure. There are considerable differences in plateau stress and Specific Energy Absorption (SEA) between the single Gyroid and IWP structures. The introduction of a hybrid design can markedly enhance the mechanical performance of these single structures. The G-IWP-1 hybrid significantly improves the structure’s SEA in loading direction I, but its effect on SEA in loading direction II is not pronounced. The plateau stresses of this hybrid structure are relatively similar in both loading directions, being notably higher than the Gyroid structure but slightly lower than the IWP structure. The G-IWP-2 hybrid shows an even more significant improvement in SEA in loading direction I, while its enhancement of SEA in loading direction II is similarly limited. This hybrid structure exhibits clear anisotropy in its plateau stress: in loading direction I, its plateau stress value is higher than that of the single structures and G-IWP-1, whereas in loading direction II, its plateau stress is lower.

Combined with the preceding analysis of the fracture modes of single and hybrid structures, it is evident that the Gyroid structure is limited by its topological characteristics and manufacturing defects inherent in PBF-LB/M-fabricated porous titanium alloy structures. This leads to premature fracture failure at multiple locations during compression, severely restricting its peak load-bearing capacity. Therefore, hybridization with the mechanically superior IWP structure can effectively improve the overall load-bearing capability. Although G-IWP-1 achieves a certain level of performance enhancement, it fails to surpass the performance limit of the IWP structure. In contrast, G-IWP-2 demonstrates excellent mechanical properties in loading direction I. Its unique 45° shear fracture failure mode confirms that the effective strengthening from the integrated IWP structure successfully suppresses the negative impacts of the Gyroid structure’s widespread fracture on plateau stress and SEA. This indicates that the spatial configuration design of the hybrid interface is key to coordinating the deformation behaviors of different unit cells. When the IWP structure acts as the primary load-bearing skeleton (as in G-IWP-2 in direction I), its excellent ductility and the Gyroid structure’s energy dissipation capability create a complementary effect. This optimizes the progressive collapse mode by suppressing fracture propagation, allowing the hybrid structure to surpass the performance limits of a single material through the directional strengthening of specific load-bearing paths.

**Fig. 11:**
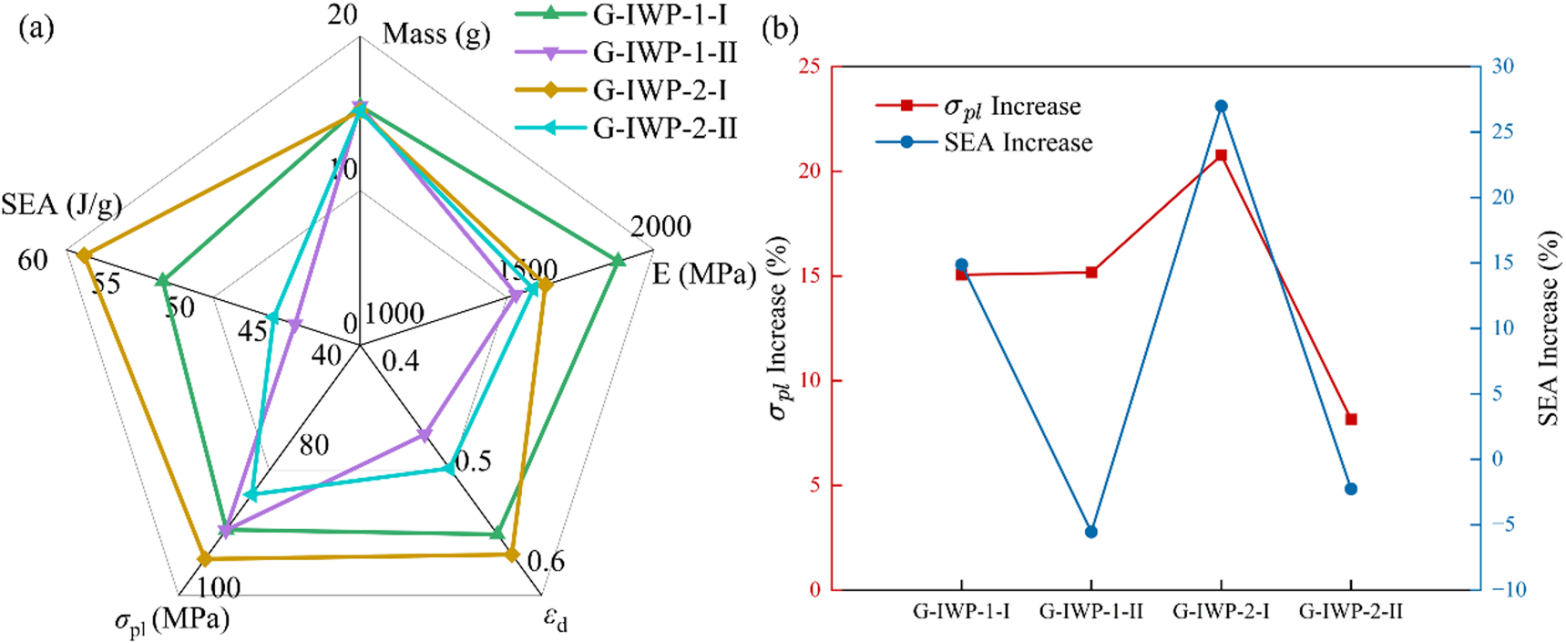
(**a**) Comprehensive performance of hybrid TPMS structures; (**b**) Comparison of plateau stress and SEA under compression and the corresponding percentage increase obtained based on.

theoretical calculations.

To provide a more intuitive evaluation and comparison of the comprehensive mechanical properties of the four specimens under static compression, a radar chart in Fig. 11(a) presents five key performance indicators: mass, elastic modulus, densification strain (*ε*_d_), plateau stress, and SEA. The chart reveals that different hybrid configurations and loading directions result in markedly distinct mechanical responses, confirming the anisotropic nature of the designed structures. Among all the configurations, G-IWP-2-I encloses the largest polygonal area, signifying its superior overall mechanical performance. Its SEA value, in particular, shows a dominant advantage, which is critical for applications that demand lightweight and highly efficient energy absorption. In contrast, while G-IWP-1-I performs well and exhibits the highest elastic modulus, it does not match the performance of G-IWP-2-I in terms of critical load-bearing capacity and energy absorption efficiency.

To further analyze the dynamic mechanical properties, the theoretical plateau stress and SEA of the hybrid TPMS structures are derived using a weighted sum model based on the contributions of their constituent homogeneous structures:

13 $${\overline {\sigma } _{{\mathrm{pl}}}}=\sum\limits_{{i=1}}^{n} {{w_i}(x) \cdot {\sigma _{pl,i}}(x)}$$

14 $$\overline {{SEA}} =\sum\limits_{{i=1}}^{n} {{w_i}(x) \cdot SE{A_i}(x)}$$

Here, ${w_i}(x)$ represents the local volume fraction of the *i*-th unit cell type in the hybrid structure. The terms ${\sigma _{pl,i}}(x)$ and $SE{A_i}(x)$ correspond to the plateau stress and SEA for a uniform structure composed entirely of that cell type.

The results confirm that the hybrid designs offer superior mechanical properties compared to their uniform counterparts, as illustrated in Fig. 11(b). Both hybrid structures exhibited an enhanced plateau stress; for instance, G-IWP-1 showed increases of 15.1% in direction I and 15.2% in direction II. The most significant enhancement was achieved with the G-IWP-2 structure under direction I loading. This configuration yielded a 20.8% increase in plateau stress and a 27.0% increase in Specific Energy Absorption (SEA), highlighting the effectiveness of the hybrid design strategy for targeted performance enhancement.

**Fig. 12:**
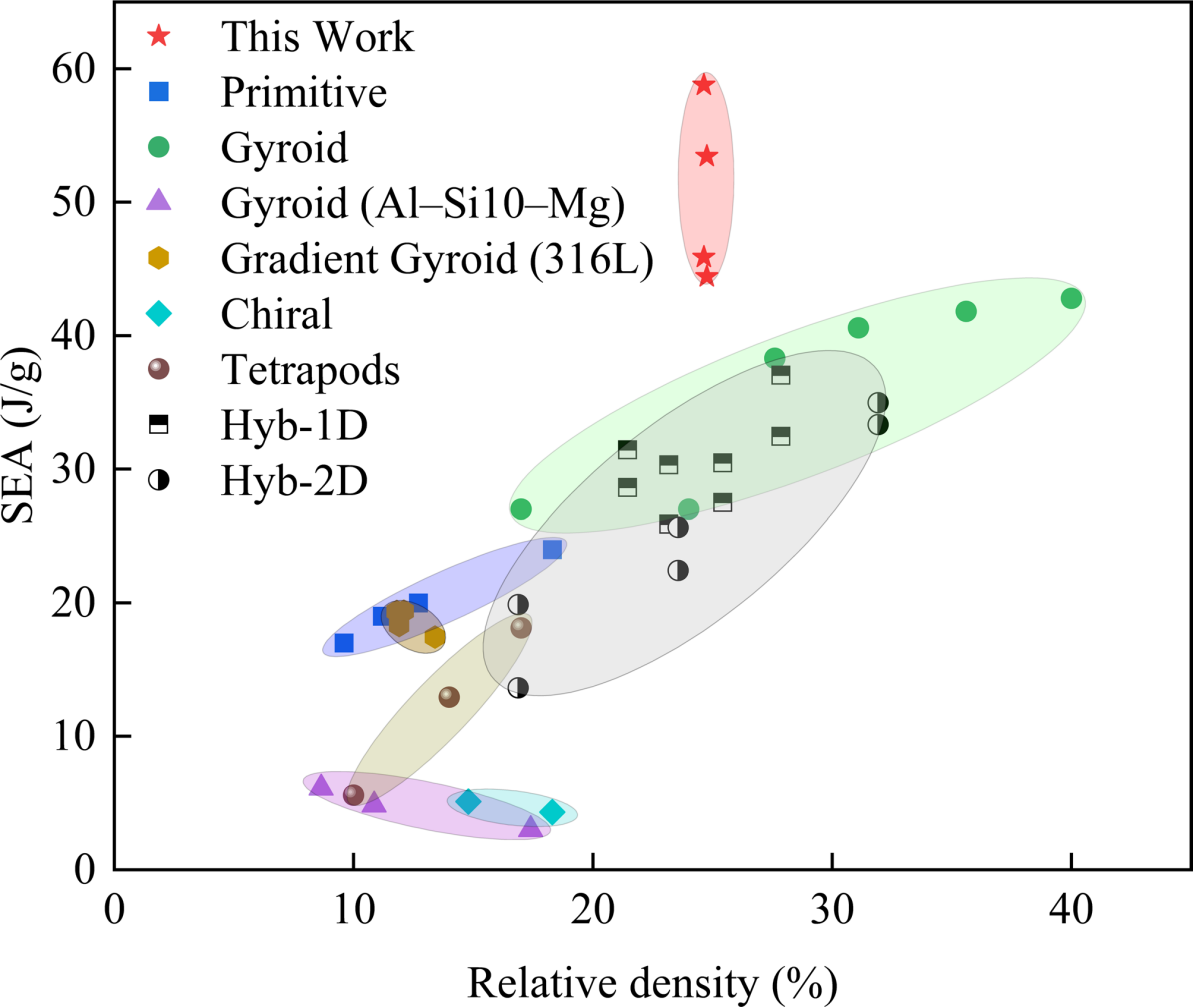
Comparison of SEA Ashby chart for various types of lattice structures.

To further evaluate the mechanical performance of the hybrid structures designed in this study, their SEA and relative density data were compared with those of various typical lattice structures reported in the literature, as shown in Fig. 12. The figure shows that the proposed G-IWP structures achieve high SEA values at a moderate relative density. Compared to the parent Gyroid structure^52,53^, which is also based on titanium alloy, the designed hybrid structures exhibit higher SEA values even at a lower relative density. When compared with other titanium alloy-based lattice structures like the P^49^, Chiral^54^, and Tetrapods^55^ structures, the G-IWP hybrids show a much higher SEA, despite having a greater relative density. The mechanical performance advantage of the designed hybrid structures is also evident when compared to Gyroid structures made from aluminum alloy^56^. This superiority is also attributed to the higher strength of titanium alloy over aluminum alloy. Similarly, the designed structures demonstrate a clear mechanical advantage over gradient Gyroid structures made from stainless steel^57^. Furthermore, when compared to other hybrid TPMS structures such as the aluminum alloy-based Hyb-1D and Hyb-2D^38^, the titanium-based G-IWP structures exhibit significantly superior energy absorption efficiency at similar relative densities.

This comparative analysis indicates that the hybrid strategy, combining Gyroid and IWP structures, effectively enhances the mechanical performance of the designs. This approach not only overcomes the performance limitations of single-topology structures but also achieves a synergistic enhancement in the core metrics of lightweight design and energy absorption. Consequently, it presents a highly promising candidate for the development of next-generation, ultra-lightweight, and high-efficiency materials for protection and energy absorption applications.

## Conclusion

In this study, we developed an in-plane hybrid design algorithm for TPMS structures, achieving a smooth transition between Gyroid and IWP topologies. The proposed hybrid designs and their uniform parent structures were successfully fabricated from Ti-6Al-4 V powder using the PBF-LB/M technique. We evaluated the surface quality of the as-built structures via scanning electron microscopy (SEM) and investigated their quasi-static mechanical properties under two distinct loading directions (I and II). Finite element simulations were also conducted to analyze the deformation behaviors, showing good consistency with the experimental data. The main conclusions are as follows:

- The Ti-6Al-4 V powder, characterized by a suitable particle size distribution and smooth morphology, proved effective for fabricating the TPMS structures. The as-built specimens feature smooth surfaces without macroscopic defects, and the deviation between designed and fabricated relative densities was under 17.77%.
- The hybrid TPMS structures based on Gyroid and IWP unit cells exhibited plateau stresses ranging from 90.87 to 99.90 MPa and specific energy absorption values between 44.43 and 58.78 J/g. Notably, the G-IWP-2-I configuration demonstrated superior overall mechanical performance.
- Compared to the uniform structures, both G-IWP hybrid designs achieved higher specific energy absorption when loaded in direction I, with improvements of 15% and 27%, respectively. In contrast, no improvement was observed in direction II.

This study focused on in-plane hybrid designs with uniform thickness to evaluate the improvement of mechanical performance and energy absorption. Future works will investigate incorporating relative density gradients to enhance energy absorption, improve failure resistance, and tailor mechanical behavior for diverse engineering applications.

## Data Availability

The datasets used and analysed during the current study are available from the corresponding author on reasonable request.
